# Imaging Efficacy of [^18^F]CTT1057 PET/CT in Patients with Biochemically Recurrent Prostate Cancer: Results from GuidePath—A Phase 3, Prospective Multicenter Study

**DOI:** 10.2967/jnumed.124.269266

**Published:** 2025-08

**Authors:** Stefano Fanti, Javier Jesus Robles Barba, Spencer Behr, Tobias Maurer, Pilar Paredes, Jochen Walz, Joan Duch, Marc Simo Perdigo, Ismini Charis Mainta, Pierre Benoit Bonnefoy, Medge Coulanges, Jun Tang, Christelle Seigne, Celine Wilke, Ana M. Catafau, Andrei Iagaru, Rahul Aggarwal, on behalf of the GuidePath study investigators, David Taieb

**Affiliations:** 1 Nuclear Medicine, IRCCS AOU di Bologna, Bologna, Italy;; 2 Nuclear Medicine, Hospital Universitari de Bellvitge, L’Hospitalet de Llobregat, Barcelona, Spain;; 3 University of California San Francisco, San Francisco, California;; 4 Department of Urology and Martini-Klinik Prostate Cancer Center, University of Hamburg-Eppendorf, Hamburg, Germany;; 5 Clinic Hospital Barcelona, Institut d’Investigacions Biomèdiques August Pi i Sunyer, University of Barcelona, Barcelona, Spain;; 6 Department of Urology, Institut Paoli-Calmettes Cancer Centre, Marseille, France;; 7 Hospital de la Santa Creu i Sant Pau, Barcelona, Spain;; 8 Hospital Universitari Vall d’Hebron, Barcelona, Spain;; 9 Division of Nuclear Medicine, Diagnostic Department, Geneva University Hospitals, University of Geneva, Geneva, Switzerland;; 10 Service de Médecine Nucléaire, CHU de Saint-Etienne, Saint-Étienne, France;; 11 Advanced Accelerator Applications, a Novartis Company, Geneva, Switzerland;; 12 Novartis Pharmaceuticals Corporation, East Hanover, New Jersey;; 13 Novartis Pharma AG, Basel, Switzerland; and; 14 Stanford University, Stanford, California

**Keywords:** biochemical recurrence, clinical trial, PET, prostate-specific membrane antigen, [^18^F]CTT1057

## Abstract

Improved diagnostic accuracy in patients with prostate cancer at first biochemical recurrence (BCR) with low prostate-specific antigen (PSA) levels is needed. This prospective study (GuidePath; NCT04838613) aimed to evaluate the imaging performance of the prostate-specific membrane antigen (PSMA)–targeted PET radiotracer [^18^F]CTT1057 to detect PSMA-positive lesions in patients diagnosed predominantly at first BCR. **Methods:** Eligible patients had a PSA of 0.2 ng/mL or greater after radical prostatectomy or an increase in PSA level of at least 2 ng/mL above nadir after radiation therapy. Patients received 370 MBq of [^18^F]CTT1057 and 150 MBq of [^68^Ga]Ga-PSMA-11 and underwent PET/CT 90 min (±30 min) and 50–100 after injection, respectively. [^18^F]CTT1057 images were assessed by 3 independent readers blinded to all clinical information. Coprimary endpoints were region-level correct localization rate (CLR) and patient-level positive predictive value (PPV) of [^18^F]CTT1057 to detect PSMA-positive lesions and were compared with a hierarchical composite truth standard (CTS). The CTS comprised 3 levels of standard-of-truth procedures (in order of priority): histopathology (CTS level 1); imaging, including at least 1 contrast-enhanced CT scan and 1 [^68^Ga]Ga-PSMA-11 PET/CT scan (CTS level 2); and a decrease in PSA level of 50% or greater 3 mo after radiation therapy (CTS level 3). For study success, the lower-bound 95% CI had to surpass 50% for region-level CLR and 20% for patient-level PPV for at least 2 of the 3 [^18^F]CTT1057 PET/CT readers. **Results:** Of 202 patients screened, 161 were evaluable for efficacy. Among these, 93.2% were experiencing their first BCR, 96.3% had received radical prostatectomy as initial definitive therapy, and baseline median PSA level was 0.4 ng/mL (interquartile range, 0.3–0.8 ng/mL). The imaging standard of truth was used for 159–160 patients (99%) across the 3 readers. Both coprimary endpoints were met. Region-level CLR ranged from 65.2% to 75.0% (lower-bound 95% CI, 53.4%–62.1%), and patient-level PPV ranged from 64.6% to 76.5% (lower-bound 95% CI, 51.8%–62.5%). **Conclusion:** [^18^F]CTT1057 met the predefined thresholds for region-level CLR and patient-level PPV in a clinically relevant patient cohort predominantly at first BCR with low PSA levels. [^18^F]CTT1057 is an accurate PSMA–targeted PET radiotracer for BCR detection.

## Visual Abstract

**Figure:**
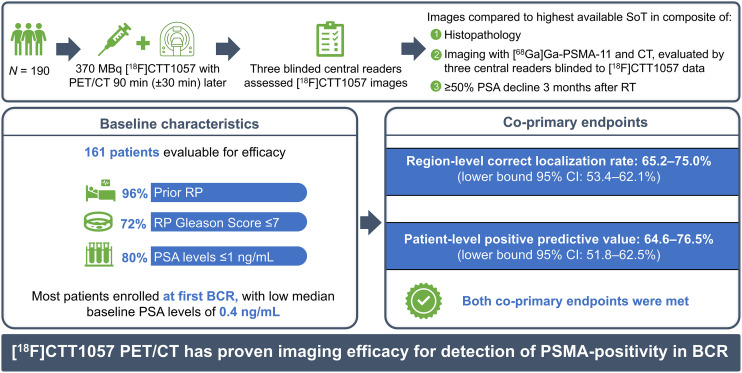

Within 10 y after primary curative-intent treatment for prostate cancer with radiation therapy (RT) or radical prostatectomy (RP), approximately 40% of patients will develop biochemical recurrence (BCR) (1,2), characterized by an increasing serum prostate-specific antigen (PSA) level. Although an increase in PSA levels can occur months or even years before clinically detectable recurrence (3), this blood-based biomarker lacks the precision required to accurately localize recurrence for further disease management (4).

Because of their low sensitivity, conventional imaging methods (e.g., bone scintigraphy, CT) are often ineffective for detecting locations of disease recurrence in patients with BCR, especially in patients with early BCR and low PSA ranges (5,6). Although multiparametric MRI can be useful for addressing local recurrence, it has limited ability to image beyond the prostatic bed without performing whole-body MRI (7). However, the utility of whole-body MRI in early BCR of prostate cancer has not been well established. Prostate-specific membrane antigen (PSMA)–targeted PET radiotracers, such as [^68^Ga]Ga-PSMA-11 (8), ^18^F-DCFPyL (9), [^18^F]PSMA-1007 (10), and [^18^F]rhPSMA-7.3 (11), have higher efficacy compared with conventional imaging approaches and are approved for PSMA-positive lesion detection in suspected BCR. Clinical trials of ^18^F-DCFPyL (CONDOR (9)) and [^18^F]rhPSMA-7.3 (SPOTLIGHT (11)) included patients with prior salvage therapy and with median PSA levels of 0.8 and 1.1 ng/mL, respectively. There remains a need to establish PSMA PET efficacy in patients at the first BCR with lower PSA levels who have met recurrence threshold, which reflects common clinical practice. As patient access to PSMA PET radiotracers in some geographic areas is limited, greater availability is needed.

[^18^F]CTT1057 (vidoflufolastat [^18^F]), a PSMA-targeted ^18^F-labeled PET radiotracer based on a phosphoramidate core (12), demonstrated high imaging accuracy for the detection of PSMA-positive lesions using histopathology as the standard of truth (SoT) in the GuideView study (13). This study, GuidePath, aimed to expand on these results by evaluating the performance of [^18^F]CTT1057 as a PET-imaging agent for detection of PSMA-positive lesions in patients with BCR.

## MATERIALS AND METHODS

### Study Design

This prospective, open-label, multicenter, single-arm, randomized, phase 3 study (NCT04838613) was undertaken across 12 sites in Europe and 1 in the United States. The protocol was approved by the institutional review board at each site, and all patients gave written informed consent. GuidePath was performed in compliance with the Declaration of Helsinki and the International Council for Harmonisation of Technical Requirements for Pharmaceuticals for Human Use E6 Guideline for Good Clinical Practice. All patients provided written informed consent.

### Patients

Patients aged 18 y or older with biopsy-proven prostate adenocarcinoma who were diagnosed with BCR after initial definitive therapy with RP or curative-intent RT (external beam or brachytherapy) were eligible. Use of prior androgen-deprivation therapy in conjunction with RT was allowed, providing an androgen-deprivation therapy washout period of more than 9 mo before study entry. BCR was defined as a PSA level of 0.2 ng/mL or greater measured at least 6 wk after RP with a second confirmatory persistent PSA level exceeding 0.2 ng/mL, according to the American Urological Association (14), or an increase in PSA level of at least 2 ng/mL above the nadir PSA observed after RT, using the American Society for Radiation Oncology-Phoenix criteria (15). After randomization of the first 20 patients, a protocol amendment on December 20, 2021, excluded patients who had received prior salvage surgery or therapy. Full inclusion and exclusion criteria are provided in Supplemental Table 1 (supplemental materials are available at http://jnm.snmjournals.org).

Between September 30, 2021, and September 4, 2023, 202 patients were screened (Supplemental Fig. 1), 190 were randomized (full analysis set), 171 received [^18^F]CTT1057 ([^18^F]CTT1057 safety set), and 161 were evaluable for efficacy (efficacy analysis set). Further details are provided in the supplemental materials.

### Imaging with [^18^F]CTT1057 and [^68^Ga]Ga-PSMA-11

Patients underwent 2 PET/CT scans (1 with [^18^F]CTT1057 and 1 with [^68^Ga]Ga-PSMA-11) at least 14 d apart. Patients were randomized 1:1 for assignment to the scan order: sequence 1 ([^18^F]CTT1057 first) or sequence 2 ([^68^Ga]Ga-PSMA-11 first).

Patients received a single intravenous injection of 370 MBq (median, 358 MBq; range, 172–405 MBq) of [^18^F]CTT1057 and 150 MBq (median, 159 MBq; range, 114–210MBq) of [^68^Ga]Ga-PSMA-11. [^18^F]CTT1057 and [^68^Ga]Ga-PSMA-11 PET/CT scans were performed 90 min (±30 min) and 50–100 min after injection, respectively. Further details are provided in the supplemental materials.

[^18^F]CTT1057 PET/CT images were assessed by 3 central independent readers (blinded to all clinical information) at the designated contract research organization. Definitions of regions and PSMA-positive uptake are provided in the supplemental materials.

### Hierarchical Composite Truth Standard

[^18^F]CTT1057 PET/CT results were compared with a hierarchical composite truth standard (CTS) with levels 1, 2, and 3 in descending order of priority: histopathology (CTS level 1); at least 1 contrast-enhanced CT scan and 1 [^68^Ga]Ga-PSMA-11 PET/CT scan (CTS level 2); and a decrease of 50% or greater in PSA level 3 mo after RT (CTS level 3) (supplemental materials) (16).

### Efficacy Endpoints

For efficacy endpoint assessments, PSMA-positive lesions reported by each of the 3 central readers who reviewed the [^18^F]CTT1057 PET/CT scan were compared with the applicable SoT, using the highest CTS level available. For comparisons with CTS level 2, anatomic correspondence between lesions reported on [^18^F]CTT1057 PET/CT scan by each reader and lesions reported on CTS level 2 imaging by consensus readers was established by an independent central reviewer.

Coprimary endpoints were region-level correct localization rate (CLR) and patient-level positive predictive value (PPV). Five regions were used for region-level endpoints: prostate, pelvic lymph node, extrapelvic lymph node, skeletal, and visceral. Further details about regions and secondary endpoints are provided in the supplemental materials. For trial positivity, the lower-bound 95% CI had to exceed thresholds set at 50% and 20% for region-level CLR and patient-level PPV, respectively, for at least 2 of the 3 readers. Statistical analyses are detailed in the supplemental materials.

## RESULTS

Table 1 presents key baseline patient characteristics. In the efficacy analysis set, the overall baseline median PSA level was 0.4 ng/mL (interquartile range, 0.3–0.8 ng/mL); most patients (80.1%) had PSA levels of 1 ng/mL or lower. In the efficacy analysis set, most patients (150/161, 93.2%) were at first BCR (89.4% had received prior RP only, and 3.7% had received prior RT only); 96.3% had received RP as initial definitive therapy. Supplemental Table 2 details the baseline characteristics, [^18^F]CTT1057 dosing, and PET image acquisition times. CTS level 1 was used for 1–2 patients across the 3 readers (1%; 1 patient with lung biopsy (visceral) and 1 patient with obturator pelvic lymph node surgery); CTS level 2 was used for 159–160 patients (99%) across the 3 readers. CTS level 3 was not deemed necessary for any patient.

**TABLE 1.** Key Baseline Clinical Characteristics

Characteristic	Full Analysis Set (*n* = 190)	Efficacy Analysis Set (*n* = 161)
Age (y)	68.0 (63.0–73.0)	68.0 (63.0–73.0)
Primary tumor clinical stage		
T2c or less	122 (64.2)	105 (65.2)
T3	7 (3.7)	4 (2.5)
T3a	32 (16.8)	28 (17.4)
T3b	9 (4.7)	7 (4.3)
T4	2 (1.1)	1 (0.6)
Tx or missing	18 (9.5)	16 (9.9)
Biopsy Gleason score		
≤6	27 (14.2)	23 (14.3)
7 (3 + 4)	64 (33.7)	59 (36.6)
7 (4 + 3)	48 (25.3)	39 (24.2)
8	30 (15.8)	25 (15.5)
9 or 10	20 (10.5)	14 (8.7)
Missing	1 (0.5)	1 (0.6)
Initial definitive therapy received		
RP	180 (94.7)	155 (96.3)
Curative-intent RT	10 (5.3)	6 (3.7)
PSA level at screening (ng/mL)*		
*n*	185	157
Median	0.4 (0.3–0.9)	0.4 (0.3–0.8)
Patients with prior RP		
*n*	176	151
Median	0.4 (0.3–0.8)	0.4 (0.3–0.7)
Patients with prior curative-intent RT		
*n*	9	6
Median	3.4 (3.2–4.2)	3.3 (2.9–4.2)
Patients who received at least 1 prior antineoplastic medication	10 (5.3)	7 (4.3)
Prior prostate cancer therapy		
RP only	162 (85.3)	144 (89.4)
RT only	10 (5.3)	6 (3.7)
RP and RT	18 (9.5)	11 (6.8)
Margin status after RP	180 (94.7)	155 (96.3)
R0	69 (36.3)	59 (36.6)
R1	86 (45.3)	73 (45.3)
Rx	25 (13.2)	23 (14.3)
Time from initial diagnosis to BCR diagnosis (mo)	33.3 (11.2–60.7)	33.3 (11.8–58.6)
Time from primary definitive therapy to BCR diagnosis (mo)	28.0 (7.4–54.4)	26.6 (7.5–53.2)

* Baseline PSA levels not evaluable in 5 and 4 patients in Full Analysis Set and Efficacy Analysis Set, respectively. Qualitative data are number and percentage. Continuous data are median and interquartile range.

**FIGURE 1.**
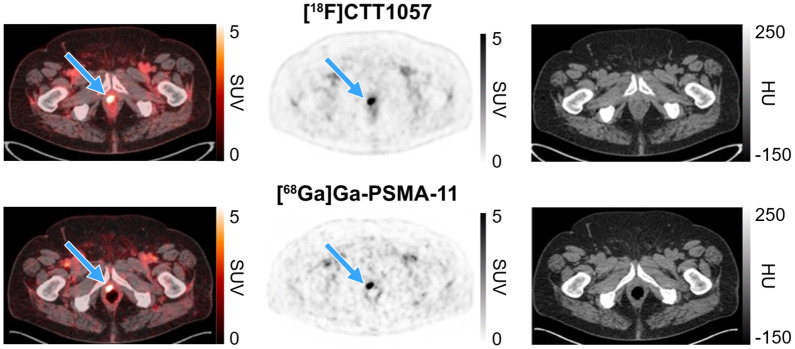
[^18^F]CTT1057 and [^68^Ga]Ga-PSMA-11 PET/CT scan images (axial slices) showing true-positive prostate bed lesion. True-positive [^18^F]CTT1057 and [^68^Ga]Ga-PSMA-11 prostate bed lesion (blue arrows) in 71-y-old patient with BCR (pT2c; Gleason score, 7 [4 + 3]) diagnosed 56 mo after initial RP (PSA level at time of PET scans, 0.49 ng/mL). HU = Hounsfield unit.

**FIGURE 2.**
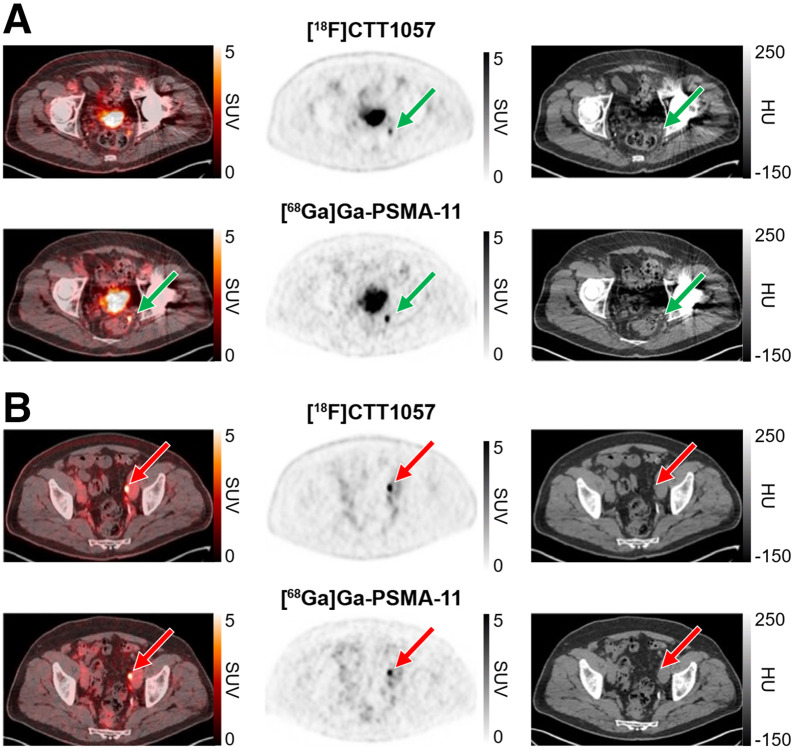
[^18^F]CTT1057 and [^68^Ga]Ga-PSMA-11 PET/CT scan images (axial slices) showing true-positive lesions in pelvic lymph node metastases. True-positive [^18^F]CTT1057 and [^68^Ga]Ga-PSMA-11 perirectal lymph node (A, green arrow) and left external iliac lymph node (B, red arrows) lesions in 71-y-old patient with BCR (pT3a; Gleason score, 7 [3 + 4]) diagnosed 67 mo after initial RP (PSA level at time of PET scans, 0.25 ng/mL). HU = Hounsfield unit.

Figures 1 and 2 show representative PET scan cases.

### Coprimary Endpoints

**FIGURE 3.**
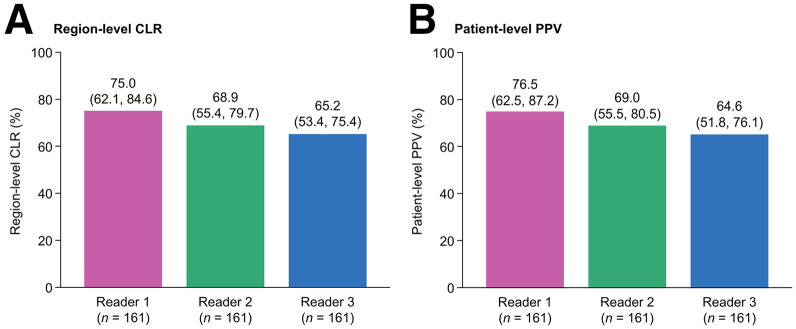
Overall region-level CLR (A) and patient-level PPV (B) of [^18^F]CTT1057 PET/CT (efficacy analysis set). Data for each reader presented as mean percentage, with 95% CI in parentheses.

Both coprimary endpoints were met. Region-level CLR ranged from 65.2% to 75.0% (lower-bound 95% CI, 53.4%–62.1%), and patient-level PPV ranged from 64.6% to 76.5% (lower-bound 95% CI, 51.8%–62.5%) (Fig. 3). Additional analyses provided results consistent with the primary analysis (Supplemental Tables 3 and 4).

Region-level CLR and patient-level PPV for patients with prior RP (*n* = 155) were consistent with the primary analysis (Supplemental Figs. 2A and 3A). In general, both coprimary endpoints showed numerically higher values with higher baseline PSA levels (Supplemental Figs. 2B and 3B). Because few patients had curative-intent RT (*n* = 6) or PSA values exceeding 1 ng/mL (*n* = 28), results for these subgroups should be interpreted with caution.

### Region-Level and Patient-Level Endpoints

Table 2 details region-level and patient-level sensitivity, specificity, accuracy, and negative predictive value. Supplemental Table 5 details the patient-level correct detection rate and detection rate of [^18^F]CTT1057 PET/CT. Supplemental Table 6 and Supplemental Figure 4 describe region-level endpoints per region.

**TABLE 2.** Imaging Efficacy of [^18^F]CTT1057 PET/CT (Efficacy Analysis Set)

	Region level (overall)			Patient level		
Parameter	Central reader 1 (*n* = 161)	Central reader 2 (*n* = 161)	Central reader 3 (*n* = 161)	Central reader 1 (*n* = 161)	Central reader 2 (*n* = 161)	Central reader 3 (*n* = 161)
Sensitivity	53.2 (42.2–63.9)	58.2 (46.7–68.8)	58.0 (47.1–68.3)	63.9 (50.6–75.8)	66.7 (53.3–78.3)	71.2 (57.9–82.2)
Specificity	98.1 (96.8–98.9)	97.1 (95.6–98.2)	96.5 (94.9–97.7)	88.0 (80.0–93.6)	82.2 (73.3–89.1)	77.5 (68.1–85.1)
Accuracy	93.7 (91.8–95.2)	93.3 (91.3–94.9)	92.7 (90.7–94.3)	78.9 (71.8–84.9)	76.4 (69.1–82.7)	75.2 (67.7–81.6)
NPV	95.1 (93.3–96.4)	95.5 (93.8–96.8)	95.4 (93.6–96.7)	80.0 (71.3–87.0)	80.6 (71.6–87.7)	82.3 (73.2–89.3)

NPV = negative predictive value.

Data are expressed as percentage, followed by 95% CI in parentheses.

### Interreader Variability and Intrareader Reproducibility

Interreader variability Fleiss κ (17) was 65.5% (95% CI, 56.8%–74.2%; Table 3). All scans were agreed upon by at least 2 readers; all 3 readers agreed on 76% of scans. Intrareader reproducibility Cohen κ (18) was 61.2%–100%.

**TABLE 3.** [^18^F]CTT1057 PET/CT Reader Variability ([^18^F]CTT1057 Safety Set)

Parameter	Central reader 1 (*n* = 171)	Central reader 2 (*n* = 171)	Central reader 3 (*n* = 171)
Total number of patients who had scan read by corresponding central reader			
Positive	56	62	69
Negative	115	109	102
Interreader variability*	65.5 (95% CI, 56.8–74.2)		
SE	0.044		
Total number of patients who had their scan read second time	19	19	19
Positive–positive	15	17	17
Positive–negative	2	0	0
Negative–positive	0	0	0
Negative–negative	2	2	2
Intrareader reproducibility^†^	61.2 (10.4–100)	100 (100–100)	100 (100–100)

* Calculated using Fleiss κ; higher interreader variability indicates higher agreement between readers.

† Calculated using Cohen k; higher values indicate higher agreement between rereads of same scan by same reader.

Data represent mean percentage, with 95% CI in parentheses.

### Change in Intended Patient Management Plans Attributed to [^18^F]CTT1057 PET/CT

After [^18^F]CTT1057 PET/CT, intended patient management plans changed for 61 patients (35.7%). Of these patients, 40 (23.4%) and 21 (12.3%) had a positive and negative [^18^F]CTT1057 PET/CT scan, respectively (Supplemental Tables 7 and 8).

### Safety

[^18^F]CTT1057 was well-tolerated and had a favorable safety profile, with no deaths or fatal events. In the [^18^F]CTT1057 safety set, adverse events were reported in 20 patients (11.7%); the most frequent were asthenia (2.9%) and increased lipase levels (1.8%) (Supplemental Table 9). Six patients (3.5%) had adverse events suspected to be related to [^18^F]CTT1057; none were grade 3 or higher (Supplemental Table 10). One patient (0.6%) had a serious adverse event (dyspnea) not related to [^18^F]CTT1057.

## DISCUSSION

GuidePath evaluated the imaging efficacy of [^18^F]CTT1057 PET for the detection of PSMA-positive prostate cancer lesions in a patient population enrolled predominantly at first BCR, after RP, with low PSA levels. Both coprimary endpoints were met.

Patients with their first BCR and low PSA levels are a commonly encountered clinical scenario requiring increased diagnostic accuracy, because they are frequently subject to misdiagnosis or underdiagnosis in clinical practice. Clinical trials of approved ^18^F-labeled PSMA PET agents ^18^F-DCFPyL (CONDOR study) (9) and [^18^F]rh-PSMA-7.3 (SPOTLIGHT study) (11) have not adequately captured this population, as patients with higher baseline median PSA levels were enrolled and those with prior salvage therapy were not excluded. In contrast, GuidePath excluded patients treated with prior salvage therapy after a protocol amendment, thus enrolling patients predominantly at first BCR. In GuidePath, most patients (96%) had undergone prior RP, compared with 85% and 78% of patients in CONDOR (9) and SPOTLIGHT (11), respectively. Consequently, the GuidePath patient cohort had a median baseline PSA level (0.4 ng/mL) lower than those of the CONDOR (0.8 ng/mL) (9) and SPOTLIGHT (1.1 ng/mL) cohorts (11), more accurately capturing this clinically relevant patient population.

The study design of GuidePath has some relevant differences compared with CONDOR (9) and SPOTLIGHT (11), including differences in the BCR patient population, the imaging component of CTS level 2, the number of regions used and methodologic differences in the estimation of region-level CLR (named region-level PPV in CONDOR and SPOTLIGHT). Despite these differences, patient-level PPV in GuidePath was between that reported in CONDOR (85%–87%) (9,19) and SPOTLIGHT (56%–72%) (20). Furthermore, region-level CLR in GuidePath was higher than region-level PPV in SPOTLIGHT (46%–60%) (11) and comparable to region-level PPV in CONDOR (67%–70%) (19). These results highlight the suitability of [^18^F]CTT1057 as an additional option for PSMA PET imaging, allowing greater patient access to accurate diagnostic information at BCR.

To our knowledge, GuidePath was the first study to use [^68^Ga]Ga-PSMA-11 PSMA PET as part of an imaging SoT. CONDOR (9) used correlative follow-up imaging (mainly ^18^F-fluciclovine PET and MRI) as the SoT for 48% of patients, whereas SPOTLIGHT (11) used ^18^F-fluciclovine PET and conventional imaging (mainly ^99m^Tc bone scan and CT) for 81% of patients. Given the high sensitivity and specificity of [^68^Ga]Ga-PSMA-11 compared with conventional imaging techniques and ^18^F-fluciclovine PET (8,21,22), [^68^Ga]Ga-PSMA-11 could overcome the limitations of these imaging approaches when used as the SoT for assessing new PSMA PET agents in settings where histopathology is not easily available. The established use of [^68^Ga]Ga-PSMA-11 PET in clinical practice and the confidence in this imaging modality for the identification of prostate cancer lesions may explain the fact that investigators did not consider it necessary to follow up with patients, neither with a 3-mo follow-up CT scan as part of the CTS level 2 when deemed necessary nor with those assessed as CTS level 3. As is expected in the BCR setting, histopathology was available as the SoT for only 1% of patients in GuidePath. However, the complementary study GuideView has demonstrated the high sensitivity and specificity of [^18^F]CTT1057 as a PET-imaging agent for detection of PSMA-positive lesions compared with histopathology as the SoT (13).

Patient-level correct detection rate and detection rate values in GuidePath were lower than those reported for ^18^F-DCFPyL (correct detection rate, 40%–43%; detection rate, 59%–66% in CONDOR and 58% in PYTHON (9,19,23)) and [^18^F]rh-PSMA-7.3 (correct detection rate, [also called verified detection rate], 51%–54%; detection rate, 68%–92% (11,20)). Lower baseline PSA levels in the GuidePath patient cohort (80% of patients had PSA levels ≤ 1 ng/mL) may account for this difference, considering that as PSA levels increase, the number of positive PSMA PET scans rises (24).

GuidePath reported substantial interreader variability and almost-perfect intrareader reproducibility according to the Landis and Koch scale (25), consistent with those reported in GuideView and for ^18^F-DCFPyL (variability, 65%; reproducibility, 81%–100%) (9) and higher than those for [^18^F]rh-PSMA-7.3 (variability, 41%; reproducibility, 46%–73%) (20). All scans were agreed upon by at least 2 readers; all 3 readers agreed on 76% of scans.

Study strengths include the imaging efficacy of [^18^F]CTT1057 to detect prostate cancer lesions in a cohort predominantly at first BCR with low PSA levels. Because PSMA PET is the most accurate imaging-based SoT for a PSMA PET agent, [^68^Ga]Ga-PSMA-11 PET was used as part of the CTS. Furthermore, [^18^F]CTT1057 showed high accuracy for detecting skeletal lesions, which may reflect an ability to reduce the unspecific bone uptake phenomenon seen with other fluorinated PSMA tracers (26,27).

Limitations of GuidePath include low patient numbers in some subgroups (i.e., patients with prior curative RT, patients with baseline PSA levels >1 ng/mL), precluding interpretation of subgroup analyses. Because PSA doubling time was not collected, patients could not be stratified by European Association of Urology risk class. Furthermore, CTS level 1 (histopathology) in GuidePath was available for a smaller proportion of patients compared with previous studies (9,11).

## CONCLUSION

GuidePath showed appropriate imaging performance of [^18^F]CTT1057 in a clinically relevant prostate cancer cohort, predominantly at first BCR with low PSA levels. Together with the high imaging efficacy shown in GuideView for the detection of PSMA-positive lesions, these results support [^18^F]CTT1057 as an additional suitable PSMA PET agent, thus enabling greater access to effective and accurate PET radiotracers.

## DISCLOSURE

This study was funded by Novartis. Stefano Fanti reports honoraria for lectures, meeting planning or advisory boards from Advanced Accelerator Applications, a Novartis company, Amgen, Astellas, Bayer, Blue Earth, Curium, Debio, GE HealthCare, Immedica, Novartis, Sofie, Telix, and United Imaging. Javier Jesus Robles Barba reports advisory board honoraria from Novartis. Spencer Behr reports honoraria from Novartis. Tobias Maurer reports speaker fees from ABX, Astellas, Bayer, Sanofi-Aventis, and Phillips; consultant fees from ABX, Advanced Accelerator Applications International S.A., Ascenian, Astellas, Axiom, Blue Earth Diagnostics, GEMoAb, Novartis, ROTOP Pharma, and Telix; and research funding from ABX, Brainlab, Intuitive Surgical, and Telix. Pilar Paredes reports speaker fees and advisory board honoraria from Advanced Accelerator Applications, a Novartis company, Astellas, and Bayer. Jochen Walz reports honoraria from Advanced Accelerator Applications, a Novartis company, Curium, Intuitive, Lightpoint, Blue Earth Diagnostics, and Telix. Marc Simo Perdigo reports speaker fees and advisory board honoraria from Advanced Accelerator Applications, a Novartis company, Astellas, Johnson & Johnson, and Bayer. Pierre Benoit Bonnefoy reports speaker fees and advisory board honoraria from Merck Sharp & Dohme. Medge Coulanges and Ana M. Catafau are employees of Advanced Accelerator Applications, a Novartis company. Jun Tang, Christelle Seigne, and Celine Wilke are employees of Novartis. Andrei Iagaru’s contribution to this publication was not part of his Stanford University duties or institutional responsibilities. He reports scientific advisory board fees from Alpha9Tx, Clarity Pharmaceuticals, and Radionetics Oncology; research grants from GE HealthCare and Novartis; consulting fees from GE HealthCare, Novartis, Progenics Pharmaceuticals, and Telix; and roles on scientific steering committees for Novartis. Rahul Aggarwal reports consulting fees and research funding to his institution from Novartis. No other potential conflict of interest relevant to this article was reported.

## Notes

KEY POINTS **QUESTION:** Can [^18^F]CTT1057 detect PSMA-positive lesions in patients at first BCR with low PSA levels? **PERTINENT FINDINGS:** In this prospective study, most patients assessed for efficacy were diagnosed at first BCR; 96.3% had received RP as initial definitive therapy, and the median baseline PSA level was 0.4 ng/mL. Both coprimary endpoints were met; region-level CLR ranged from 65.2% to 75.0% (lower-bound 95% CI, 53.4%–62.1%) and patient-level PPV ranged from 64.6% to 76.5% (lower-bound 95% CI, 51.8%–62.5%). **IMPLICATIONS FOR PATIENT CARE:** [^18^F]CTT1057 could expand access to PSMA radiotracers for the detection of prostate cancer lesions in patients at first BCR, a population frequently misdiagnosed or underdiagnosed in clinical practice.
